# Consumption of Oleic Acid During Matriphagy in Free-Living Nematodes Alleviates the Toxic Effects of the Bacterial Metabolite Violacein

**DOI:** 10.1038/s41598-020-64953-x

**Published:** 2020-05-15

**Authors:** Kyoung-hye Yoon, Tong Young Lee, Je-Hyun Moon, Seong Yeol Choi, Yun Ji Choi, Robert J. Mitchell, Jin Il Lee

**Affiliations:** 1 Division of Biological Science and Technology, College of Science and Technology, Yonsei University, Mirae Campus, Gangwon-do, South Korea; 2 School of Life Sciences, Ulsan National Institute of Science and Technology, 50 UNIST-gil, Ulsan, South Korea; 3 Present Address: Department of Physiology, Mitohormesis Research Center, Yonsei University Wonju College of Medicine, Wonju, Gangwon-do South Korea

**Keywords:** Ecological genetics, Behavioural ecology, Behavioural genetics, Bacterial toxins, Animal behaviour

## Abstract

Maternal behaviors benefit the survival of young, contributing directly to the mother’s reproductive fitness. An extreme form of this is seen in matriphagy, when a mother performs the ultimate sacrifice and offers her body as a meal for her young. Whether matriphagy offers only a single energy-rich meal or another possible benefit to the young is unknown. Here, we characterized the toxicity of a bacterial secondary metabolite, namely, violacein, in *Caenorhabditis elegans* and found it is not only toxic towards adults, but also arrests growth and development of *C. elegans* larvae. To counteract this, *C. elegans* resorted to matriphagy, with the mothers holding their eggs within their bodies and hatching the young larvae internally, which eventually led to the mothers’ death. This violacein-induced matriphagy alleviated some of the toxic effects of violacein, allowing a portion of the internally-hatched young to bypass developmental arrest. Using genetic and pharmacological experiments, we found the consumption of oleate, a monounsaturated fatty acid produced by the mother, during matriphagy is partially responsible. As such, our study provides experimental evidence of why such a drastic and peculiar maternal behavior may have arisen in nematode natural habitats.

## Introduction

Animals go to extraordinary lengths to increase the survival and reproductive fitness of their young. Matriphagy, which occurs when organisms consume their own mother, is an extreme adaptation that is observed in some animals. For instance, in the spider species *Diaea ergandros* and *Amaurobius ferox*, mothers willingly and, in the case of *A. ferox*, actively invite their young to devour them^1^. Although matriphagy eliminates the mother’s ability to provide for and protect their brood, or even reproduce again, the nutritional benefit to the young outweighs these deficiencies^1^.

Predators of bacteria are a major factor controlling the death and survival of bacteria in their native environments^7^. In response, microbes have evolved diverse methods to combat predation, including the synthesis of toxic secondary metabolites^8^. One such compound that decreases the survival of microbial predators is violacein^9,10^. Violacein is a bis-indole secondary metabolite produced in a diverse array of bacteria^2^, where its production is regulated by quorum-sensing mechanisms^3,4^. Violacein is toxic to many organisms including Gram-positive bacteria^4^, fungi^5^, and protozoa^6,7^. Violacein production, which is an energy expensive process due to being a derivative of two tryptophan molecules^8^, is considered an adapted defense mechanism to protozoal grazing of bacteria^9^. Animals are not free from the effects of violacein either: the free-living hermaphroditic nematode *Caenorhabditis elegans* is also susceptible to violacein toxicity. *C. elegans* feeding on a strain of *E. coli* engineered to produce violacein had decreased survival^10^, and learned to avoid violacein-producing *E. coli*^11^.

*C. elegans* can be found in nature in rotting fruits and vegetation^12^ where large and diverse populations of bacteria can be found around and even inside the worms^13^. In prosperous times, mother worms consume bacteria and lay hundreds of eggs, who themselves will hatch and grow up to be hermaphroditic mothers within a few days. However, in these natural environments *C. elegans* are often exposed to many types of stress, including heat, overcrowding, starvation and pathogenic strains of bacteria, fungi and viruses^14^. One of the common strategies that adult *C. elegans* mothers employ during times of stress is to hold their eggs inside their bodies and allow the larvae to internally hatch. In a form of matriphagy the larvae then consume the mother from the inside, killing the mother and forming a “bag of worms”. Eventually the young break out of the hard remaining cuticle “bag” and enter the surrounding environment. It has been suggested that internal hatching is a way to provide enough nutrition to the young to allow them to reach dauer, an alternative developmental stage that offers long-term protection to the young worm in times of duress^15^.

Here, we report that violacein is not only toxic to the adult animal, but has multiple effects across the life of the worm including complete developmental arrest in young larvae as well as intestinal defects and internal hatching of larvae in adult animals. Interestingly, we observed that the internally hatched young from these experiments often bypassed larval arrest into adulthood. Using genetics and pharmacology, we found internally hatched larvae have a significant fitness advantage over larvae from eggs laid in violacein. We also observed adding crushed worms ameliorates the developmental effects of violacein and identified oleic acid as one of the possible nutritive factors that aid larvae when they are exposed to violacein. Finally, we reveal differential toxic effects of violacein in several free-living nematodes, yet found a conserved role for the positive effects of oleic acid. Taken together, we demonstrate that matriphagy in *C. elegans* confers reproductive fitness advantages to the worm in a bacterially-induced toxic environment.

## Materials and Methods

### Nematode culture and strains

Worms were grown and maintained at 20 °C on Nematode Growth Medium (NGM) plates seeded with *E. coli* OP50 as described previously^16^. Strains used for this study, *N2*, *unc-54*, and *nhr-49*, as well as *C. briggsae* (AF16), *C. remanei* (EM464), *P. pacificus* (PS312) were obtained from the *Caenorhabditis* Genetic Center (University of Minnesota, USA). *Pelodera sp.* was isolated from the wild.

### Preparation of OP50-vio

OP50 *E. coli* strain was transformed with pCOM10vio, a violacein expression plasmid that was originally constructed by Dr. Xin-Hui Xing^17^. The violacein operon (*vioABCDE* genes) within this plasmid is under transcriptional control of the *alkB* promoter.

To prepare the OP50-vio lawns on NGM plates^16^, approximately 50 μl of a log-phase LB culture of OP50-vio was pipetted on to the center of the NGM agar media. This agar contained kanamycin (25 µg/ml) to maintain the pCOMvio plasmid. The plate was incubated overnight at 37 °C to allow the bacteria to initially grow and then incubated for 2 days at 20 °C to induce violacein production. The plates were UV-irradiated at 0.72 J/cm^2^ using a UV crosslinker (Ultra-violet Products, LTD., UK) and stored at 4 °C until use. For plates containing FUDR (5-fluoro-2′-deoxyuridine), 120 uM FUDR was added to the NGM agar prior to growing *E. coli* OP50-vio.

For the liquid culture experiments, overnight LB cultures of OP50-vio were sub-cultured into fresh LB media with kanamycin and grown to log phase. Violacein was induced by adding 0.05% octane to the culture and incubating it overnight at 20 °C with shaking 200 RPM. After growing, the bacterial cells were pelleted, then washed twice with sterile water. The final pellet was resuspended in S-basal media and stored 4 °C until use.

### Survival assay

#### Solid

Synchronized Day 1 adult worms were placed on NGM plates with either OP50 or OP50-vio. Survival was assessed every 1–3 days.

#### Liquid

All survival assays involving fatty acid supplementation were carried out in liquid assays so that bacteria is not required to grow in the supplemented media, which could alter violacein production. For these tests, 10–25 worms at Day 1 adult stage were placed in each well of a 96 well plate containing S-basal media supplemented with 120 μM FUDR. OP50 or OP50-vio was then added for food. Worm survival was assessed every 1–3 days. More food was added as needed.

### Development assay

#### Solid

Synchronized L1 worms were pipetted onto the bacterial lawns. Body lengths were measured on day 4.

Liquid culture (fatty acid supplementation): Synchronized L1 worms were pipetted into each well, which contained S-basal media and a supplemental fatty acid. Body length was measured on days 4 and 6, by placing worms in an agarose padded glass slide with coverslip. To assess growth of larvae after internal hatching, a few adult worms were placed inside the well and the lengths of the progeny were measured after 10 days. To measure their lengths, the worms were placed on an agarose padded glass slide and covered with a coverslip. They were then imaged using an Olympus compound microscope with DIC filters.

### Fatty acid supplementation

In order to preclude the possibility that the addition of fatty acids to NGM plates may have on induction of violacein, we tested the effect of fatty acid supplementation in only liquid assays. All the fatty acids tested were purchased from Sigma-Aldrich (USA), with sodium salts used for stearic acid and oleic acid. Each fatty acid stock was prepared according to Deline *et al*., 2013^18^.

Juglone and acrylamide, both purchased from Sigma-Alrich (USA), were added at the indicated concentrations. The juglone stock was always prepared fresh and at 1000x concentration. For acrylamide, a 30% 30:1 acrylamide:bis-acrylamide solution was used.

### Quantification of bacteria in the *C. elegans* intestinal lumen

L4, or adult worms, were placed on OP50-vio lawn as prepared above. After 1–4 days the accumulation of purple bacteria in the gut lumen was observed. Worms were washed twice in a small volume of sterile water directly on the NGM plates then moved to 1.5 ml Eppendorf tubes with 200 μl of autoclaved water. The worms were broken open using a motorized pestle, and the resulting suspension was diluted (10^−2^ and 10^−3^) using sterile water and spread out on a LB plate containing kanamycin. After incubation at 30 °C overnight, the number of colonies were enumerated and used to calculate the colony forming unit (CFU) in the gut of each worm.

## Results

### Violacein toxicity in larva and adult *C. elegans*

**Figure 1:**
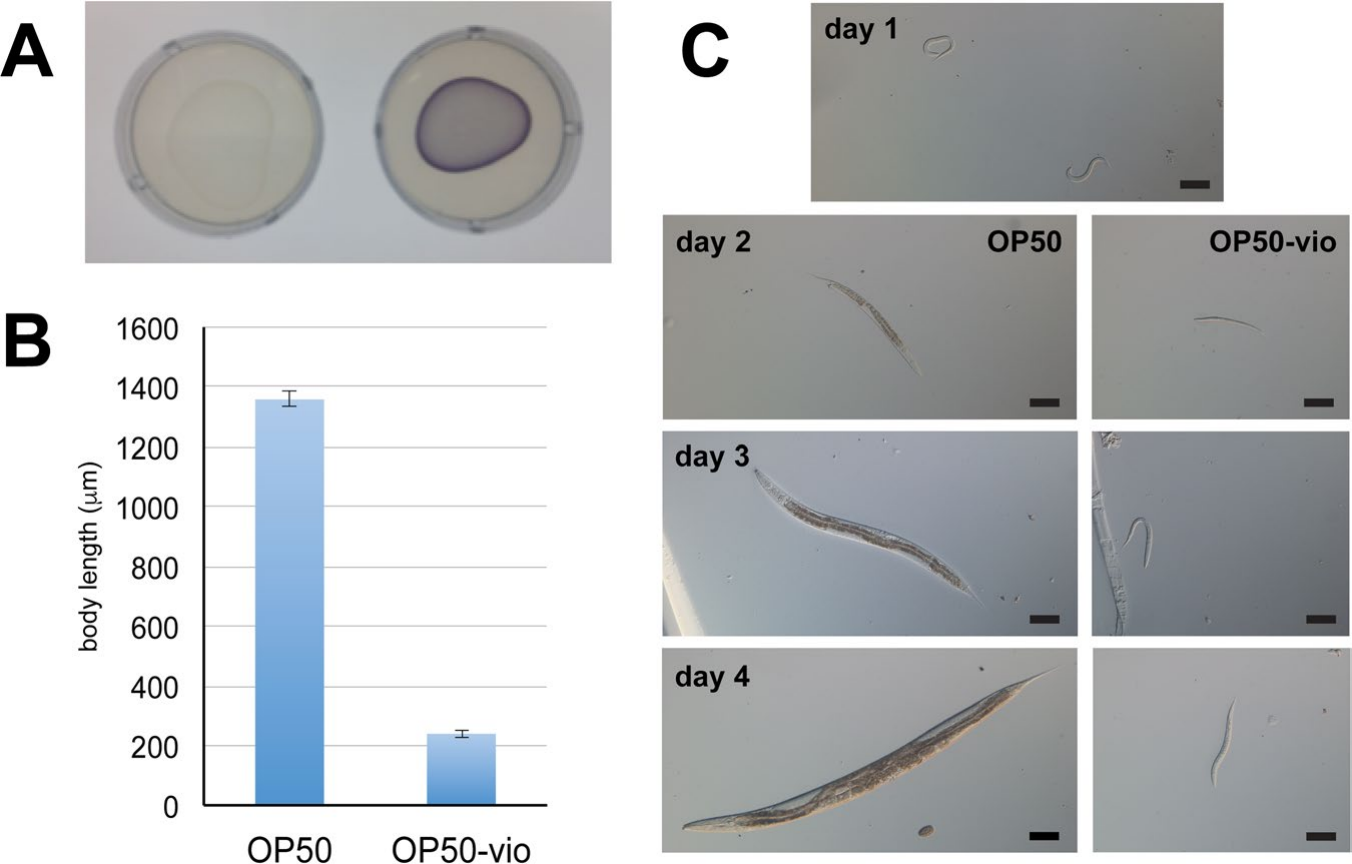
Violacein stunts the growth and development of *C. elegans*. (**A**) Lawn of *E. coli* strain OP50 (left) and violacein-expressing OP50 (OP50-vio, right). (**B**) Body length of worms grown on OP50 and OP50-vio from L1 larvae stage for 4 days. (**C**) Development of worms grown on OP50 and OP50-vio. Day 1 image show L1 synchronized worms that has never been fed. Scale bar = 100 μm.

To gain a deeper understanding of the effects of violacein on *C. elegans* physiology and development, we made a violacein-producing *E. coli* strain OP50 by transforming it with pCOM10vio plasmid, which expresses the five genes of the violacein operon under the PalkB promoter, commonly used in *E. coli* for inducible expression^17^ (henceforth called OP50-vio). Expression of violacein was induced at 20 °C, with a purple hue visually apparent when OP50-vio is grown on standard nematode growth media (NGM) (Fig. 1A). Based on previous observations with DH5α (unpublished observation), OP50-vio is expected to produce comparable levels of violacein as bacteria found in the wild^19^. Wild-type N2 worms grown on normal OP50 proceed through several larval stages from L1 to L4 and grow in size before becoming an adult worm after 4 days, at which point they laid their own eggs. However, worms grown on OP50-vio showed little growth and eventually experienced a developmental arrest in early larval stages (Fig. 1B,C). Thus, violacein has a profoundly negative impact on *C. elegans* growth and development.

**Figure 2:**
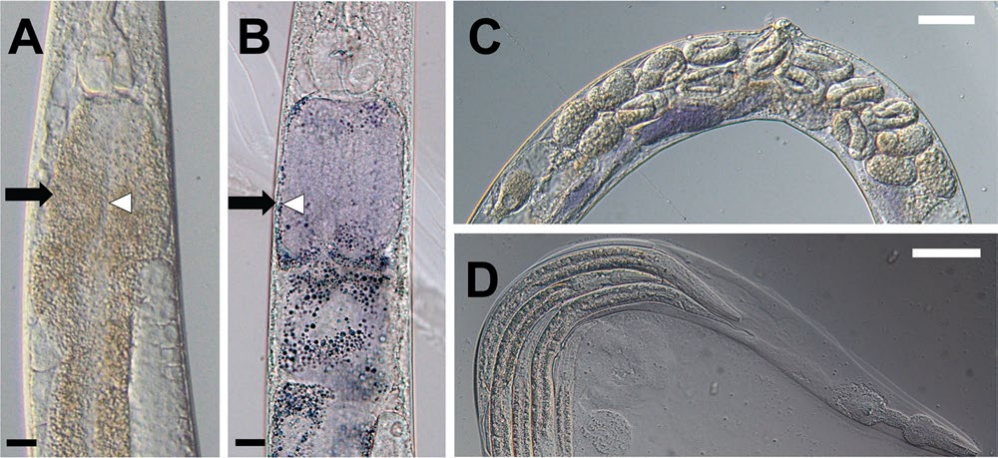
Violacein induces multiple defects in the adult hermaphrodite mothers. (**A**,**B**) Adult nematodes exposed to violacein develop intestinal defects. (**A**) shows OP50-fed worms and (**B**) shows worms fed OP50-vio. Intestinal walls (black arrows) are thinner and lumen width (white arrows) is wider in OP50-vio exposed adult animals. (**C**) Adult hermaphrodite mothers in OP50-vio display late-stage eggs within their body. (**D**) Internally hatched larvae worms inside a dead mother’s remaining cuticle in OP50-vio. Black scale bar indicate 20 μm, white scale bar 50 μm.

Violacein also imparted serious consequences to the adult worm. When adults were exposed to violacein overnight, we observed its accumulation in the intestine, along with distension of the gut lumen and general intestinal morphology defects (Fig. 2A,B), which is similar with previous reports^10^. Worms showed a smaller and shriveled appearance in general.

In addition to these intestinal effects, consumption of violacein inhibited the mother worm from laying her eggs, who instead held them within her body. Under normal developmental processes, *C. elegans* oocytes are fertilized internally, and the mother lays the eggs through her vulva when the embryo reaches between the 16 to 32-cell stage. However, in violacein-treated mothers late stage embryos can be seen inside the mother worms’ body (Fig. 2C), and eventually we observed internal hatching of the embryos in many of the mothers (Fig. 2D). In this phenotype, commonly seen when gravid *C. elegans* mothers are exposed to adverse environmental conditions^15,20–25^, some of the embryos that are not laid will hatch internally, and the larvae will begin to feed and grow within the mother’s body. This results in the death of the mother and a “bag of worms” phenotype in which larvae are surrounded by the mother’s remaining cuticle (Fig. 2D). Under such conditions, the larvae continue to grow and finally break open the mother’s cuticle before being released into the surrounding environment.

**Figure 3:**
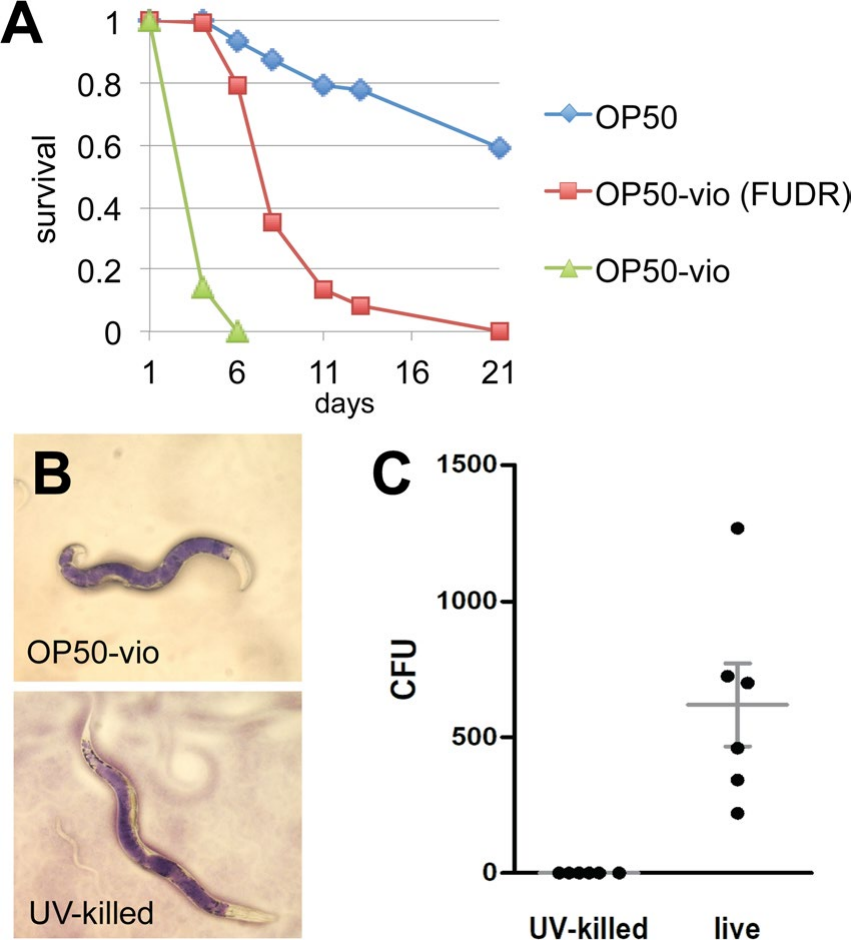
Violacein shortens lifespan independent of internal hatching and intestinal colonization. (**A**) Survival of *C. elegans* adults in OP50-vio treated with or without FUDR treatment. *C. elegans* worms normally die within 5–6 days on OP50-vio due to internal hatching, but worms treated with the sterilizing agent FUDR can survive up to 20 days. Representative graph from 3 trials; n ranges from 50 to 136. (**B**) Violacein accumulates in the intestine of both OP50-vio and UV-killed OP50-vio cultured worms, indicating defect in passing contents out of the intestine, rather than active colonization of bacteria. (**C**) UV-killed OP50-vio does not colonize the intestine. Colony counts of bacteria inside the intestinal lumen of each worm with either OP50-vio or UV-killed OP50-vio (n = 6).

Previous reports demonstrated that violacein decreased the *C. elegans* lifespan significantly^10,11^. However, neither of those studies distinguished whether violacein toxicity or internal hatching was directly responsible for worm death. Consequently, we evaluated *C. elegans*’ survival on OP50-vio with or without addition of 5-fluoro-2′-deoxyuridine (FUDR), a chemical that prevents internal hatching by inhibiting cell division and subsequent development of the embryos. As shown in Fig. 3A, the presence of FUDR significantly increased survival of the worms, suggesting that internal hatching is a major cause of violacein-induced death (Fig. 3A). This is not to say that this is the only form of toxicity, though, as the survival rates with FUDR treatment were still much lower for worms grown on OP50-vio than normal OP50 (Fig. 3A). Thus, violacein-induced death is due to both internal hatching and general toxicity.

Violacein-producing *E. coli* was previously shown to colonize and accumulate in the worm intestine, which would contribute to toxicity to the worm^10^. To evaluate whether colonization of OP50-vio is required for death, we UV-irradiated OP50-vio to kill the bacteria. When we fed adult worms the UV-killed OP50-vio, we still observed accumulation of violacein pigment in the intestine (Fig. 3B), even though we confirmed that the accumulated bacteria were dead (Fig. 3C). Moreover, the survival rate of *C. elegans* when provided UV-killed OP50-vio was indistinguishable from the live OP50-vio (Fig. S1). This demonstrates that at least when expressed in OP50, death is a result of violacein itself rather than the colonization of violacein-producing bacteria in the gut.

### Internal hatching ameliorates the developmental effects of violacein

In our evaluation of violacein toxicity, an interesting observation was made. Although violacein completely arrests larval development so that none of the larvae can reach adulthood (Fig. 1), we noticed that in some of the old OP50-vio plates in which adult worms were placed more than a week before, the adults had already died; however, a small portion of adult worms from the next generation had grown up. This was confounding as we already showed that young larvae cannot develop and survive in violacein (Fig. 1B,C). Since we knew that *C. elegans* mothers in violacein allow their larvae to hatch internally (Figs. 2C,D), we hypothesized that internal hatching conferred some benefit to allow the larvae to survive and grow in the presence of violacein.

**Figure 4:**
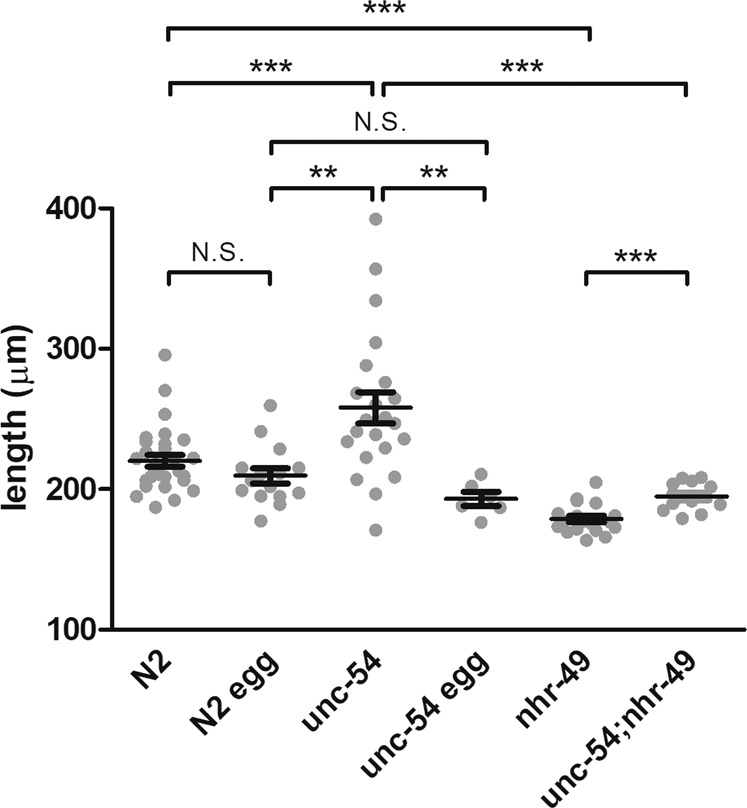
Nutrients obtained from internal hatching ameliorate the developmental effects of violacein. Length of worms measured after 10 days in violacein. N2 indicates wild type mothers placed in OP50-vio, resulting in both externally-laid and internally-hatched larvae. N2 egg indicates eggs that were collected from N2 mothers and directly placed into OP50-vio. Young of *unc-54* mutant mothers, which are all internally hatched, grew significantly larger than wildtype. This effect was abolished in isolated eggs from *unc-54* that hatched outside the mother. Young of *nhr-49* mutant mothers, which produce low levels of oleic acid, do not grow as well even when 100% of the larvae internally hatch (*unc-54;nhr-49*). Each dot represents average values of different trials. **Indicates p < 0.01, ***p < 0.001.

To test whether the presence of the mother worm could mitigate violacein’s effect on development, we placed either young adult mother worms or just their eggs directly in OP50-vio for 10 days and compared the growth of the resulting larvae. We found larvae from the laid eggs (N2 egg) did not grow as well as those that grew in the presence of their mothers (N2), but the difference was very subtle and statistically insignificant (Fig. 4). Because it takes several hours for the worms to stop laying eggs in response to violacein, most eggs are laid and only a small portion of young will hatch internally. To ensure that most of the larvae hatch internally within the mother’s body, we measured the growth of young from *unc-54* mutant mothers in violacein. Mutants of the myosin heavy chain gene *unc-54* lack functioning muscle, including the vulva muscle, meaning 100% of the embryos develop within the mother and hatch internally^26^. Although they are severely defective in movement, they grow quite normally to adulthood, albeit slightly shorter in length (Fig. S3). The larvae from *unc-54* mutant mothers, on average, grew better in the presence of violacein than larvae from either the wild-type eggs or wild-type mothers (Fig. 4). Particularly, some of the *unc-54* larvae grew substantially longer, *i.e*., to adulthood or near adulthood, which was rarely seen in N2 larvae and never observed in N2 eggs (Table 1). To verify that this effect is due to internal hatching, we prepared eggs from *unc-54* worms to hatch outside the mother in OP50-vio. Eggs hatched outside the mother displayed stunted growth comparable to N2 eggs (Fig. 4). This indicates that internal hatching, which is induced in the presence of violacein, provides a significant benefit for some of the larvae against the bacterial toxin.

**Table 1:** Advanced larvae development in violacein.

Body length	N2 egg	N2	*unc-54*
> 400 µm	0%	0.43%	2.72%
>500 µm	0%	0%	0.82%
Sample size	N = 515	N = 1391	N = 368

### Oleic acid improves developmental survival in violacein

**Figure 5:**
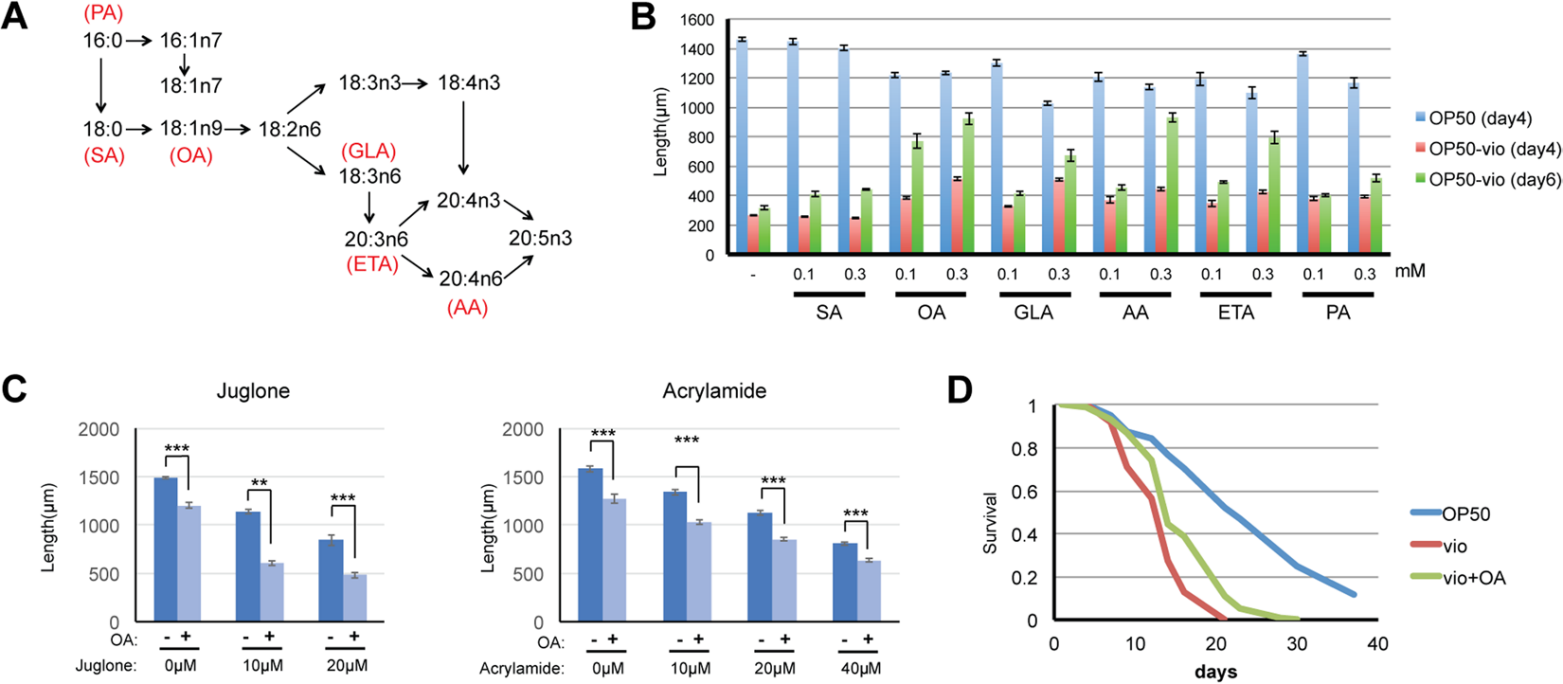
Exogenous supplementation of oleic acid and some PUFAs improve worm growth in OP50-vio. (**A**) Synthesis pathway of mono-and poly-unsaturated fatty acid from saturated fatty acid in C. elegans (modified from Watts and Browse, 2002). SA = stearic acid, OA = oleic acid, GLA = γ-linoleic acid, AA = arachidonic acid, ETA = eicosatetraenoic acid, PA = palmitoleic acid (**B**) Worms supplemented with individual fatty acids showed improvements in growth in OP50-vio for 4 days (red) or 6 days (green) at either 0.1 mM or 0.3 mM concentration exogenous fatty acid. Growth in supplemented OP50 is shown in blue. Oleic acid had the most effect, and PUFAs ETA and AA also showed improvement in day 6. All values for fatty acid supplemented samples for OP50-vio 4 days and 6 days have p values <0.001 compared to unsupplemented control (−), except for SA. All values within the same fatty acid are statistically significant (p < 0.001). 30 to 90 worms were tested for each condition. (**C**) Oleic acid does not mitigate toxicity from the oxidative stressor juglone and neurotoxin acrylamide. Graph shows average of 3 trials. 26 to 43 worms tested for each condition. Statistical significance calculated by Students’ T-test. (**D**) Oleic acid improves survival of adult worms in OP50-vio. All fatty acids were supplemented at 0.3 mM unless otherwise indicated. Graph show representative results from 3 trials. **Indicates p < 0.01, ***p < 0.001.

Previous reports have shown that matriphagy by internally hatched larvae provides the young with a small nutritional benefit in a food-depleted environment^15,25^. This small amount of food is enough to allow some of the L1 young to develop sufficiently to reach the L2 dauer stage, an alternative developmental stage that allows worms to survive in adverse environments for long periods of time. However, we observed here that matriphagy ameliorates the L2 developmental arrest of a bacterial toxin allowing a portion of the young to reach near adulthood. We wondered then what nutritional component from the mother’s body was responsible for the improved growth in the presence of violacein. Among the possible candidates, we focused on lipids. Fats, both saturated and unsaturated, play integral roles in development, reproduction and aging in the worm^27^. When compared to the lipids found in bacteria, *C. elegans* lipids in general tend to contain longer chain fatty acids that reach up to 20 carbon fatty acids^27^. *C. elegans* also synthesizes polyunsaturated fatty acids from saturated and monounsaturated fatty acids, and the enzymes responsible for the elongation and desaturation steps have been well-studied (Fig. 5A)^28^.

To determine whether fatty acids may be a nutritive factor that mitigates the developmental effects of violacein, we tested a number of saturated and unsaturated fatty acids *C. elegans* can intrinsically synthesize. We analyzed growth of *C. elegans* larvae simultaneously treated with violacein and the following fatty acids: 18-carbon (SA, 18:0) saturated fat stearic acid, its monounsaturated derivative oleic acid (OA, 18:1n9), and the polyunsaturated fatty acids (PUFA) γ-linoleic acid (GLA, 18:3n6), arachidonic acid (AA, 20:4n6), eicosatetraenoic acid (ETA, 20:4n3) and palmitoleic acid (PA, 16:1n7). We found that after four days of growth in violacein the saturated fatty acid had no effect; however, some of the unsaturated fatty acids could partially attenuate violacein’s toxic effects on growth (Fig. 5B). After 6 days in violacein, untreated worms remain arrested in development, but it was clear now that worms incubated with the monounsaturated oleic acid had a vast improvement in growth. To a lesser extent, omega-6 PUFAs arachidonic acid and eicosatetraenoic acid also had positive effects that became more apparent after day 6. Although oleic acid improves development of worms in violacein, it was previously shown to have toxic effects^29^. Indeed, when we increased the concentration of oleic acid further, any enhancements were counterbalanced by its negative effects on growth (Fig. S2).

Oleic acid may be alleviating toxicity by either a violacein-specific mechanism or by a general toxicity response. We investigated the latter possibility by testing whether oleic acid can mitigate other forms of toxicity. Juglone is a chemical that induces toxic reactive-oxygen species (ROS). Increasing the concentration of juglone results in higher levels of worm death, but oleic acid treatments did not increase survival (Fig. 5C). Acrylamide is a known neurotoxin, and high levels of acrylamide kill worms^30^. Oleic acid, though, cannot mitigate the effects of acrylamide either (Fig. 5C). We speculate then that the protective nature of oleic acid against violacein is not functioning via a general stress or toxicity response mechanism.

In addition to improving the worm’s development, we asked whether oleic acid also improves the survival of adults exposed to violacein. In conditions preventing internal hatching by FUDR, the same concentration of oleic acid that improved development also improved the survival of adult worms (Fig. 5D). Thus, oleic acid improves the worm’s ability to overcome the toxic effects of violacein during both larval development and survival.

### Oleic acid consumed during matriphagy alleviates the effects of violacein

Although an exogenous application of oleic acid mitigated violacein-induced growth arrest, we wondered whether oleic acid produced in the body of the mother worms and consumed by the young after internal hatching is sufficient to alleviate violacein toxicity. We decided to pursue this question genetically. Mutants of the *nhr-49* that encodes a nuclear hormone receptor homologous to the mammalian PPARα show decreased levels of oleic acid^31^. We wondered, then, if internally hatched young from *nhr-49* mutants could still bypass the toxic effects of violacein since their mothers produce less oleic acid. We found that although adult *nhr-49* worms were not inherently smaller than N2 adult worms (Fig. S3), larvae born to *nhr-49* mutant mothers in violacein were significantly smaller than larvae from wild-type mothers (Fig. 4). To ask whether larvae internally hatched in mothers deficient in oleic acid could grow in violacein, we tested *nhr-49;unc-54* double mutant larvae. Compared to how internal hatching visibly improved growth in the presence of violacein (N2 vs *unc-54*), in the background of the *nhr-49* mutation, internal hatching resulted in only a slight mitigation of their growth defects (*nhr-49* vs *unc-54;nhr-49*) (Fig. 4). Hence, we conclude that maternal oleic acid consumed by larvae after internal hatching mitigates violacein toxicity. We note that there is, however, a statistically significant improvement in growth in the internally hatched *nhr-49* mutants. This may be due to the physical protection provided by the mother’s cuticle in the early larvae, or due to other nutrients that also contribute to their improved growth in violacein.

### Differential toxicity of violacein in species of free-living nematodes

**Figure 6:**
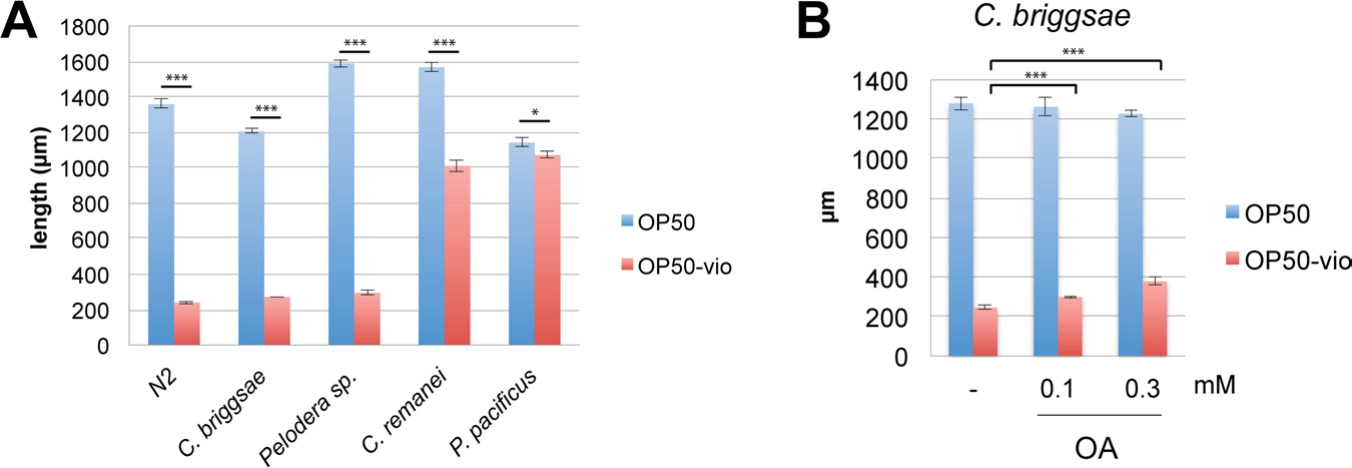
Differential toxicity of violacein in other nematode species. (**A**) Growth of various nematode species in OP50 and OP50-vio from the L1 larvae stage. Similar to *C. elegans*, *C. briggsae* and *Pelodera sp.* are sensitive to violacein whereas *C. remanei* development is slightly affected by violacein and *P. pacificus* are unaffected by violacein. (**B**) Growth of *C. briggsae* in violacein is improved by 0.1 mM and 0.3 mM oleic acid (OA) supplementation. ***Indicates p < 0.001, *p < 0.05. Significance determined by Students’ T-test. All graphs show average results of 3 trials.

Violacein production is associated with quorum-sensing, a form of pheromonal communication in bacteria. The purple pigment is an excellent indicator that bacterial communities are communicating with one another and preparing to form biofilms. Along with violacein, quorum-sensing induces bacteria to produce hundreds of other secondary metabolites that can have toxic effects to organisms that come in contact with them. Indeed, non-pathogenic bacterial biofilms induce a stress response in *C. elegans*^32^, and the pathogenicity of *Chromobacterium violaceum* towards *C. elegans* is dependent on quorum-sensing mechanisms^33^. In light of this, we asked whether violacein toxicity is a conserved mechanism for bacteria to evade bacteriovorous nematode predation. To evaluate this, we examined whether violacein could affect growth in four free-living nematode species, *C. briggsae, C. remanei, Pelodera sp*., and *Pristionchus pacificus*. Surprisingly, we found that only two of these species, *C. briggsae* and *Pelodera sp*., showed severe developmental arrest similar to *C. elegans* (Fig. 6A). Larval development in *C. remanei*, which is most closely related to *C. elegans*, was only partially compromised, and *P. pacificus* development was completely unaffected. From this we can conclude that the toxicity of violacein depends on the species of nematode.

### Mitigation of violacein toxicity by oleic acid is conserved among nematodes

We demonstrated that *C. elegans* and two other species of nematodes were susceptible to violacein. Could oleic acid also ameliorate violacein toxicity in other nematode species? We examined this question in *C. briggsae*, a closely related *Caenorhabditis* member. We found that violacein toxicity to *C. briggsae* larvae was quite strong, even compared to *C. elegans* (Fig. 6A,B). Interestingly, exogenous oleic acid could partially ameliorate even these severe effects (Fig. 6B). Thus, we demonstrate here that oleic acid’s beneficial affects against violacein toxicity is conserved in nematodes.

## Discussion

Predators of bacteria, mainly protozoa and nematodes, are a main factor controlling the death and survival of bacteria in their native environments^34^. Microbes have adapted diverse techniques to combat predators, including the synthesis of toxic secondary metabolites^35^. Previous work has shown that the secondary bacterial metabolite violacein shortens *C. elegans* lifespan and increases bacterial infection in the intestine^10^. In addition to these, we have shown here that violacein itself can stunt *C. elegans* development and growth and inhibit egg laying, the latter resulting in internal hatching of larvae within the mother and her eventual death by matriphagy. Even worms that escape death by matriphagy due to FUDR treatments are still subject to the toxic effects of violacein and show decreased lifespans. We show that worms fed UV-killed OP50-vio, in which no intestinal colonization occurs, still experience the same level of violacein accumulation in the intestine and death rates. The exact mechanism of how violacein kills *C. elegans* remains uncertain. Interestingly, we found that only some nematodes are vulnerable to violacein, and even closely related nematodes such as *C. remanei* and *P. pacificus* are mostly immune to its effects. It is known that *C. remanei* and *P. pacificus* are generally more resistant to toxins than *C. elegans*^36,37^, a characteristic that also holds true for violacein (Fig. 6). Understanding how these nematodes are protected may help us understand the mode of violacein’s toxicity.

Violacein production, although an energetically expensive process^8^, is an effective defense against bacterial grazing by protozoa^9^. Similarly, killing of adult *C. elegans* by violacein is a powerful bacterial defense against a bacteriovorous predator, but stunting the development and killing all the predator’s progeny is devastating to the nematode’s reproductive fitness and evolution. Just as bacteria have adapted techniques to combat predation, nematodes have adapted the peculiar behavior of holding live embryos within their own body so they hatch internally. This is a well-known phenomenon in nematodes when environmental conditions are not optimal^15,38^. One obvious benefit of internal hatching in this case is that the larvae are physically shielded from violacein because of the mother’s cuticle while the larvae feeds on the mother’s body, giving them a “head start” in development. Indeed, previous studies have shown that internal hatching aids starved L1 larvae in reaching the late L1/early L2 stage necessary for dauer formation, which allows worms to survive longer in harsh environmental conditions^15,25^. Internal hatching provides a physical shield from the outside environment for the young larvae, while matriphagy provides a small amount of nutrition to the developing young. This nutrition may help some larvae reach dauer, an alternate developmental stage in nematodes to survive harsh conditions. While this gives the worms a better chance of moving to a gentler environment, it seems that it does not give them any benefit afterwards: according to Chen and Caswell-Chen (2004), when the internally hatched progeny are returned to a standard laboratory diet of OP50, they do not display any advantage in fecundity or lifespan compared to larvae that hatched outside the mother’s body^15^.

Here, we propose that an additional benefit exists for internal hatching and matriphagy, namely that in the presence of certain toxins, such as violacein, internal hatching not only provides a temporary physical shield from the toxins, but allows the young to access to nutrients that increases their resistance to the toxin. This results in improved larvae development and growth, and, in some instances, allows the young to reach adulthood. Moreover, we found that one of these nutritional components is the monounsaturated fatty acid oleic acid. Although some PUFAs that can be derived from oleic acid, such as arachidonic acid and ETA, also mitigate the negative effects of violacein somewhat, they seem to require higher concentrations and a longer time to produce the same effect (Fig. 5B). However, because PUFAs cannot be obtained through a bacterial diet but are produced by the worms themselves, it is possible that these may also contribute to improved resistance to violacein during matriphagy. The beneficial effects of oleic acid to health are well known^39^, but in *C. elegans* oleic acid can actually stunt growth^29^ (Fig. 5B). However, in the presence of violacein oleic acid has a clearly positive effect on worm fitness.

How can oleic acid alleviate violacein toxicity? Two previous studies reported the role of fatty acids in immunity. Gamma-linolenic acid (18:3n6) and stearidonic acid (18:4n3) were shown to be required to maintain *C. elegans*’ basal immunity against *Pseudomonas aeruginosa* through phosphorylation levels of the MAPK protein PMK-1, and that mutants that cannot produce these fatty acids are more susceptible to infection^40^. A more recent study showed that oleic acid is required for the induction of immune defense genes in response to several pathogens, including *P. aeruginosa*, *S. marcescens*, and *E. faecalis*, and worms unable to synthesize oleic acid are hyper-susceptible to infection^41^. Considering that violacein is produced by pathogenic bacteria, oleic acid, in addition to functioning in immunity, may also help detoxify the compounds produced by those bacteria. As immunity genes and detoxification genes are often induced together^42^, oleic acid may be acting upstream of both immunity and detox pathways. Interestingly, oleic acid had no ameliorating effect on juglone or acrylamide toxicity, which suggests it only acts on certain detox genes. Moreover, the fact that oleic acid has similar effects in other nematodes suggests a conserved evolutionary mechanism.

One interesting note is that *C. elegans* may be more prone to internal hatching than *C. briggsae* or *C. remanei* because it lays eggs at a later stage (16–32 cell stage) than *C. briggsae* or *C. remanei*, which lay eggs at the 2 cell stage (wormatlas.org). Thus, the fact that *C. elegans* mothers prefer to hold eggs within their bodies longer than other nematodes may confer a fitness advantage for *C. elegans* when faced with toxins and environmental hazards.

## Supplementary information

Supplementary Information.
