# Supplementary material for: Consumption of Oleic Acid During Matriphagy in Free-Living Nematodes Alleviates the Toxic Effects of the Bacterial Metabolite Violacein
Source: Sci Rep. 2020 May 15;10:8087. doi: 10.1038/s41598-020-64953-x (PMC7229185; doi:10.1038/s41598-020-64953-x)
Supplement: Supplementary file 1 — Supplementary Information. [file 41598_2020_64953_MOESM1_ESM.docx]

**Supplementary Figures**

**Consumption of Oleic Acid During Matriphagy in Free-Living Nematodes Alleviates the Toxic Effects of the Bacterial Metabolite Violacein**

Kyoung-hye Yoon^1,3^, Tong Young Lee^1^, Je-Hyun Moon^1^, Seong Yeol Choi^2^, Yun Ji Choi^1^, Robert J. Mitchell^2, *^, and Jin Il Lee^1, *^

^1^ Division of Biological Science and Technology, College of Science and Technology, Yonsei University, Mirae Campus, Gangwon-do, South Korea

^2^ School of Life Sciences, Ulsan National Institute of Science and Technology, 50 UNIST-gil, Ulsan, South Korea

^3^ Current address: Department of Physiology, Mitohormesis Research Center, Yonsei University Wonju College of Medicine, Wonju, Gangwon-do, South Korea

^*^ Corresponding authors:

Robert J. Mitchell, 50 UNIST-gil, Ulsan 44919, South Korea, [esgott@unist.ac.kr](mailto:esgott@unist.ac.kr), tel: +82-52-217-2513, fax: +82-52-217-2509;

Jin Il Lee: Mirae 304, 1 Yonseidae-gil, Wonju, Gangwon-do 26493, South Korea, [jinillee@yonsei.ac.kr](mailto:jinillee@yonsei.ac.kr), tel: +82-33-760-2249, fax: +82-33-760-2183


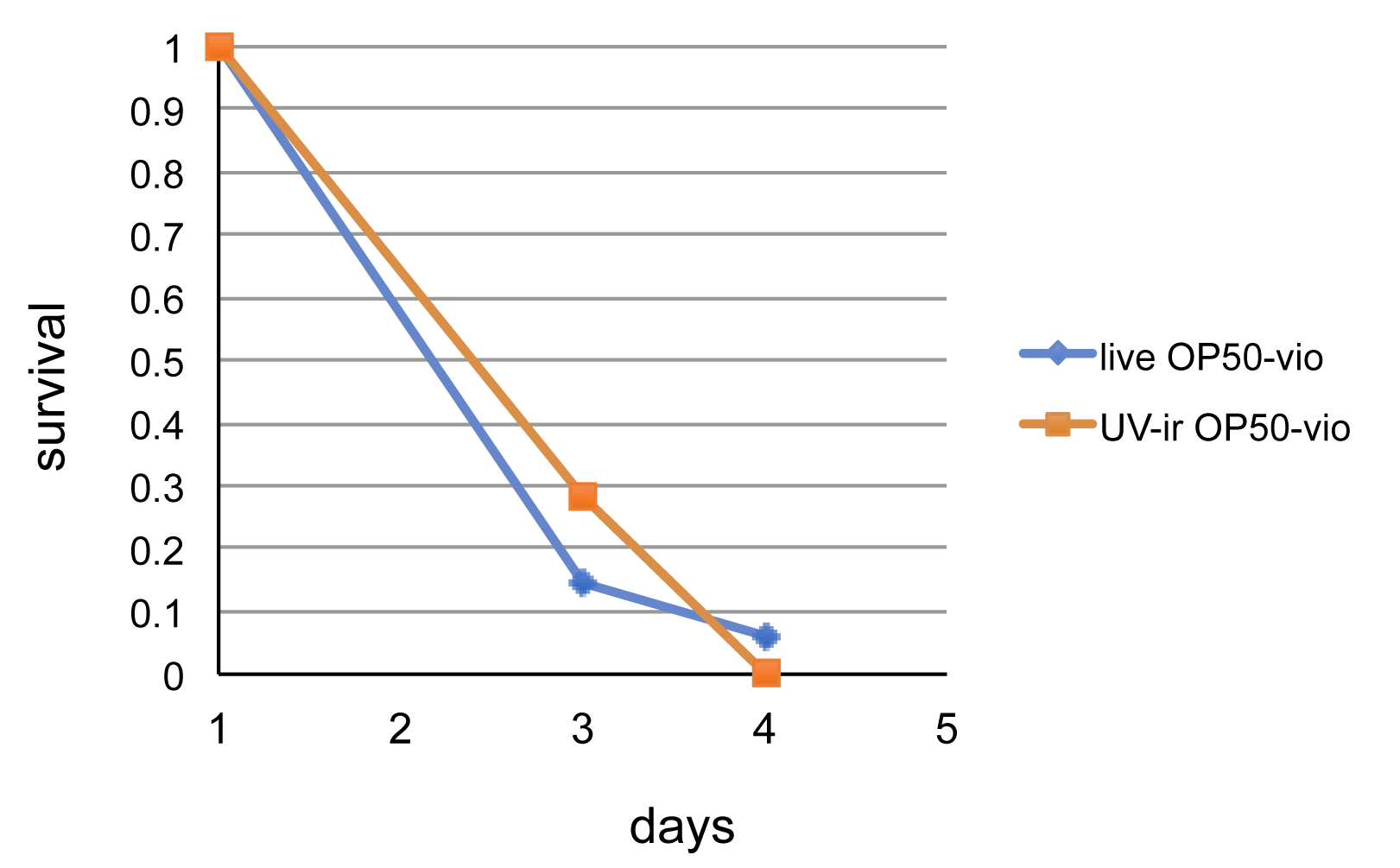


**Figure S1. No difference of potency observed between live and UV-killed OP50-vio.** Survival of adult animals exposed to live and UV-killed OP50-vio. Graph shows a representative trial.


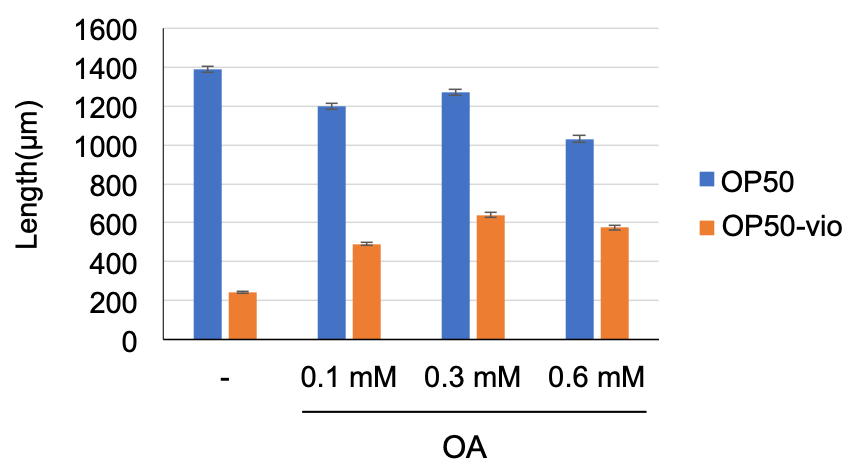


**Figure S2. Higher concentration of oleic acid does not further improve growth in violacein.** High concentrations of oleic acid (0.6 mM) results stunted or delayed growth in OP50.


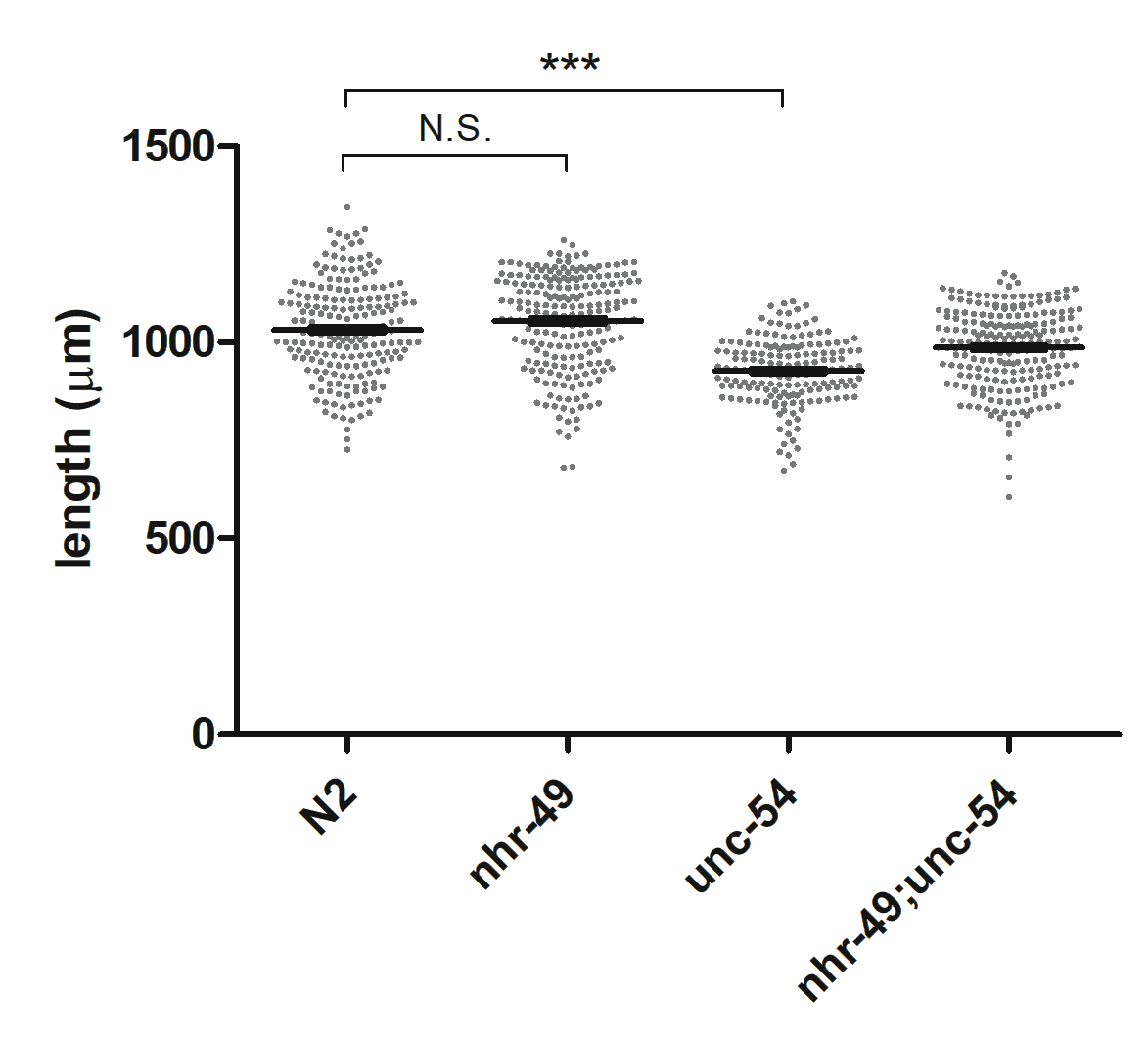


**Figure S3. Length of *nhr-49* worms does not significantly differ from N2 in OP50-fed conditions.** Body length after normal growth to adulthood in OP50 bacteria in N2 strain, *nhr-49*, *unc-*54, and *nhr-49;unc-54* double mutants. Dots represent a single worm, line shows the average and bars indicate S.E.. Statistical significance determined by Students’ T-test.
